# Super-resolution imaging with nanopipettes

**DOI:** 10.1038/s44303-025-00108-9

**Published:** 2025-09-22

**Authors:** Steffan Møller Sønderskov, Lasse Hyldgaard Klausen, Sebastian Amland Skaanvik, Xiaojun Han, Mingdong Dong

**Affiliations:** 1 Interdisciplinary Nanoscience Center (iNANO), Aarhus University, Aarhus, Denmark; 2 Dansk Fundamental Metrologi-Danish National Metrology Institute, Hoersholm, Denmark; 3 Department of Chemistry, Western University, London, ON Canada; 4 State Key Laboratory of Urban Water Resource and Environment, School of Chemistry and Chemical Engineering, Harbin Institute of Technology, Harbin, China; 5 Laboratory of Biosensors and Bioelectronics, Institute for Biomedical Engineering, ETH Zurich, Zurich, Switzerland

**Keywords:** Biophysics, Chemistry, Engineering, Nanoscience and technology

## Abstract

Uncovering structural information of biological systems at the nanoscale is vital for understanding their dynamics and function. Nanoscale imaging techniques that obtain structural information down to the single-molecule level under physiologically relevant conditions and without affecting the fragile structure of biomaterials are limited. Thus, the realization of such techniques is highly attractive, especially within the biological sciences. Nanopipette-based imaging using scanning ion conductance microscopy (SICM) fulfills these requirements, but resolution limitations and artefact formation hinder obtaining accurate structural information on the scale comparable to the pipette tip. Here, we present a novel technique, super-resolution SICM (SR-SICM), based on image deconvolution using simulated pipette point-spread functions. The technique is demonstrated on different types of nanostructures, where it surpasses the lateral resolution limit of SICM and mitigates imaging artefacts considerably. SR-SICM is applicable to any SICM dataset through user-friendly downloadable software promoting the possibility of single-molecule studies on a routine basis.

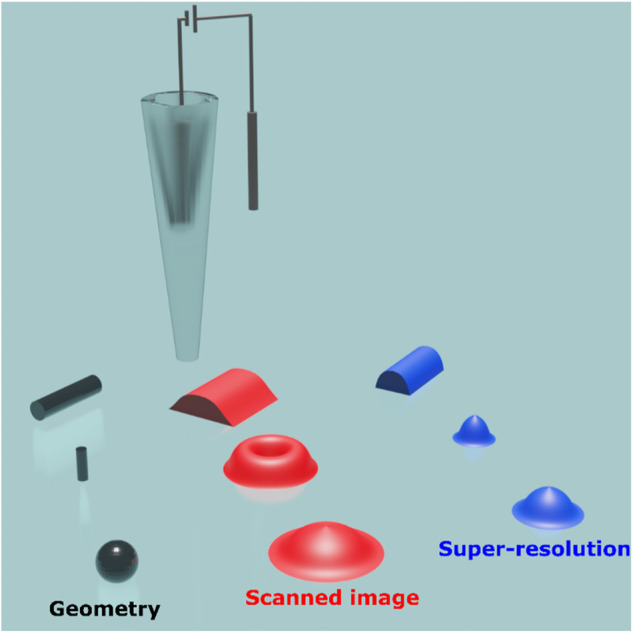

## Introduction

Microscopy techniques that offer nanoscale resolution under physiological conditions are invaluable tools for gaining insight into the dynamics and functions of biological systems down to the single-molecule level. Reliable and high-resolution imaging of biological molecules and specimens is dependent on the measurements being carried out under native-like conditions, where especially the use of a physiological buffer, optimal temperature and pressure are vital^1^. Light microscopy and fluorescence microscopy in particular have traditionally been powerful techniques for sub-micron studies^2,3^. However, fluorescence microscopy requires the presence of photostable fluorescent molecules, which are usually artificially incorporated by use of labeling techniques^4^. This can lead to disadvantages such as poor recognition capabilities or interference with the dynamics of the system^5,6^. Furthermore, fluorescence microscopy is still limited by the diffraction limit of light in the lateral dimension, although recently optical super-resolution techniques have been developed to improve the resolution beyond the diffraction limit. These super-resolution techniques are generally based on the alignment of multi-image sequences, such as for stochastic optical reconstruction microscopy^7^ and photoactivated localization microscopy^8^, or single-image deblurring methods such as deconvolution^9^.

Electron microscopy and scanning probe microscopy (SPM) are powerful imaging techniques that can overcome the limitations of optical microscopy. Electron microscopy offers high resolution but is confined to vacuum imaging. Meanwhile, SPM techniques such as atomic force microscopy (AFM) can perform high-resolution imaging under physiological conditions^10^. AFM relies on the physical interaction between a sharp tip and the sample, resulting in a lateral resolution determined by the contact area between tip and sample, normally in the range of a few nanometers for samples that are not atomically flat^11^. Recently, though, the fundamental resolution of AFM has been substantially improved by applying localization algorithms to multi-image sequences, with an improvement in resolution of up to a factor of 5^12^. However, the technique still relies on a physical interaction between a probe and the sample, which is known to affect the sample structure^10^. In light of the successful utilization of optical super-resolution techniques, the development of a nanoscale microscopy technique based on a non-contact scanning probe method that has been enhanced by applying optical image-enhancing methodology can become a valuable nanoscale imaging tool.

Micro- and nano pipettes are powerful and versatile sensing tools used for the detection of single molecules, monitoring processes in biological systems and as scanning probes for studying various surface properties down to the nanoscale^13–18^. Non-contact topographical imaging with glass pipettes at the micro- and nanoscale is accomplished by passing an ionic current through a glass pipette and simultaneously measuring the pipette position and current occlusion, as the pipette is gradually moved to the proximity of a surface. A constant bias potential (*U*) is applied between quasi-reference electrodes placed inside and outside the pipette, respectively. The ionic current (*I*) is given by the total resistance (*R*_T_), which can be divided into a constant pipette resistance (*R*_P_), and a variable access resistance (*R*_acc_). *R*_acc_ is often approximated as a function of the pipette inner (*r*_i_) and outer (*r*_o_) radii, the resistivity of the solution (*ρ*) and the pipette-sample distance (*d*)^19,20^:

1 $$I\left(d\right)=\frac{U}{{R}_{T}}=\frac{U}{{R}_{p}+{R}_{{acc}}}$$

2 $${R}_{{acc}}=\frac{\rho }{2\pi d}{\text{ln}}\left(\frac{{r}_{o}}{{r}_{i}}\right)$$

This technique is referred to as scanning ion-conductance microscopy (SICM)^16^ and has been utilized as a powerful nanoscale tool for studying the topography of fragile and soft samples non-destructively^21^, mapping surface charge on the nanoscale^22–24^ and combined with other techniques for versatile analytical platforms for surface studies^25–27^. The imaging capabilities of SICM are determined by the ionic flux profile near the pipette orifice^28^. SICM imaging properties have been explored in detail, and based on this, several fundamental advantages and disadvantages of SICM have been reported^22,28–30^. The mechanism of SICM provides a unique non-contact imaging opportunity that comes at the expense of low lateral resolution and the emergence of numerous imaging artifacts. Enhancing the fundamental sensing capabilities of SICM by improving the resolution and mitigating imaging artifacts can lead to a powerful new microscopy technique for high-resolution imaging of water-submersed biological systems and biomolecules.

In this study, we present a novel technique, which we term super-resolution scanning ion-conductance microscopy (SR-SICM), capable of providing high-resolution imaging of water-submersed structures with non-contact. To overcome the fundamental imaging capabilities of SICM, we first provide a fundamental study into the ionic flux profile at the pipette orifice, which we term the sensing zone. In the presence of a point structure, we obtain the point-spread function (PSF) of SICM, which we utilize with a post-processing methodology to enhance the imaging capabilities of SICM.

The performance of SR-SICM is first determined on basic and complex geometrical shapes and then on SICM images of biological nanostructures, including lipid bilayers, protein fibrils and DNA nanostructures. In all these cases, SR-SICM is shown to be a highly versatile and powerful technique for achieving significantly higher image resolution, while also mitigating SICM-related image artifacts. SR-SICM is applicable regardless of pipette geometry and imaging conditions and can be applied to virtually any SICM image. However, a potential drawback facing users utilizing this technique is the necessity for in-house finite-element method (FEM) software and sufficiently powerful computing resources in order to obtain custom pipette PSFs. To accommodate this issue, we have developed a user-friendly downloadable software application containing a library of PSF profiles. Here, users can obtain PSFs based on custom pipette geometries and perform SR-SICM on imported SICM images. The powerful imaging capabilities displayed by SR-SICM, along with its straightforward use, can lead to the advancement of pipette-based imaging of nanoscale structures and dynamics down to the single-molecule level.

## Results

### Enhancing the fundamental image formation of SICM

A microscope spatially visualizes geometric features or other properties of an object, which can otherwise not be observed. During this process, image degradation is present due to artifacts caused by imperfections or physical limitations of the imaging instrument^31^. This applies to SICM, which suffers from numerous imaging artifacts due to its non-contact operation and a worse lateral resolution due the complex shape of the pipette^12,29,32^.

Image processing techniques are acknowledged procedures for recovering or enhancing an image and have been traditionally utilized for optical microscopy^33^ and more recently for SPM methods^12,31,34–37^. One such powerful method is image deconvolution, where an observed image, *g*(*x, y*) is a convolution of the true image, *f*(*x, y*), and the microscope PSF, *h*(*x, y*), which is the imaging response to a point structure. The relation is formulated in Eq. 1, where * is the convolution operator.

3 $$g(x,y)=f(x,y)\ast h(x,y)$$

Determining the PSF of a microscope and applying Eq. 3 can restore a low-quality image to one of higher quality, thereby mitigating microscope artifacts and moving beyond the physical resolution limit. Applying this approach to pipette-based imaging methods, such as SICM, requires knowledge of the pipette PSF determined by the pipette geometry and imaging conditions^32^. The geometry of the pipette is described by the inner (*r*_i_) and outer (*r*_o_) pipette radius, along with the inner half angle (*θ*). Experimentally, geometric information of the pipette can be acquired by various methods, such as measuring pipette current and applying either FEM or analytical methods to approximate the inner and outer pipette radii^38,39^, or using scanning electron microscopy or transmission electron microscopy^39^.

However, obtaining a characteristic PSF of SICM is troublesome because of the broad range of pipette geometries and imaging parameters^32,39,40^. An alternative approach would be to determine the PSF under experimental conditions, which would be time-consuming and require custom-produced micro- or nanofabricated structures and thereby unpractical. Thus, the utilization of deconvolution methods for SICM image enhancement requires a method for obtaining custom and precise PSFs effectively. To overcome this obstacle, we propose to gather profiles of the PSF by FEMs, which are powerful computational tools which have been applied for understanding the fundamental properties of pipette-based imaging and measurements^29,32,38^. By applying these methods, a custom pipette geometry can be constructed, and a detailed understanding of the fundamental image formation process can be obtained under specific imaging conditions. Hereafter, deconvolution algorithms can be applied to obtain a super-resolution image.

**Fig. 1:**
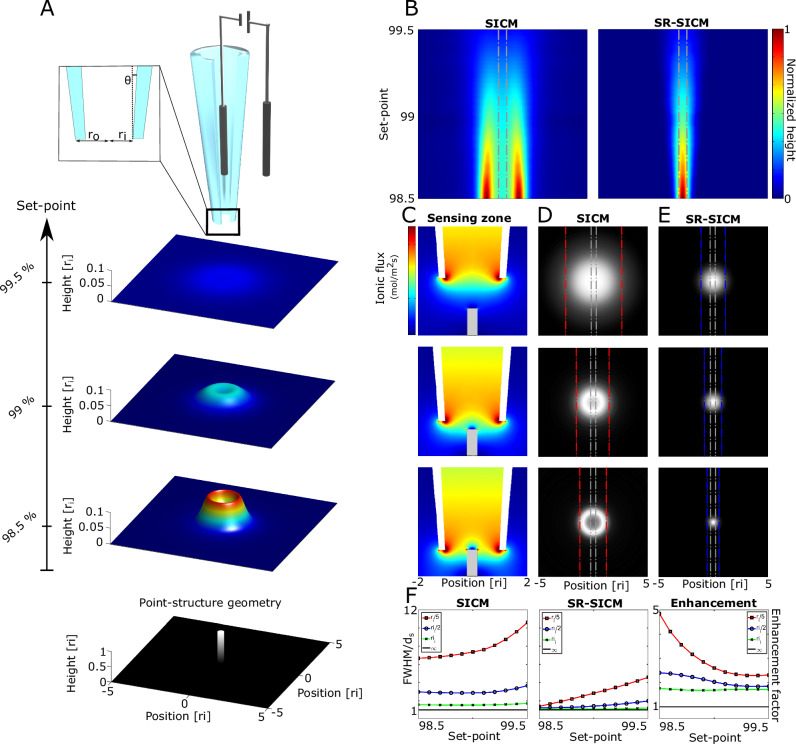
Principle of SR-SICM. **A** Principle of SICM and imaging output of a point structure. **B** Line-profile plots at different set-points for SICM and SR-SICM showing the substantial improvement of the fundamental imaging capabilities. **C** Sensing zone in the vicinity of a point structure along with the (**D**) SICM and **E** SR-SICM imaging output (point-structure geometry is highlighted by the gray stippled lines, while the FWHM of the imaging profile is visualized by red and blue stippled lines for SICM and SR-SICM, respectively). **F** Plots of FHWM_SICM_/d_s_ and FHWM_SR-SICM_/d_s_ at different imaging set-points along with the quantification of the SR-SICM imaging enhancement.

The principle of SR-SICM, and its improvement over regular SICM is shown Fig. 1. First, a model is constructed of a pipette and a point structure (details found in Supplementary Fig. 1). The model is scalable, and the dimensions are therefore given in terms of the inner radius, *r*_i_, which will normally be in the range of 10–100 nm. Following this, simulations of the SICM imaging output are performed at different set-points (Fig. 1A). The fundamental imaging profiles of SICM and SR-SICM are shown in detail from cross-sections of resulting images at different imaging set-points before and after deconvolution (Fig. 1B). The imaging properties of SICM are determined by the profile of the ionic flux in the vicinity of the pipette orifice, which we term the sensing zone. When a geometry, such as a point structure, progressively enters the sensing zone (Fig. 1C) by decreasing the imaging set-point, the flux profile is increasingly modified due to the partial occlusion of ionic current. This is studied in detail by simulating the imaging output at 3 common imaging set-points (99.5, 99, and 98.5%) (Fig. 1D). Here, the point structure appears significantly larger than the actual geometry (dashed gray lines) for high set-points due to the occurrence of large diffusional broadening. However, this broadening rapidly decreases as the set-point is decreased, leading to the point structure appearing as a solid ring mirroring the pipette geometry.

In the corresponding SR-SICM images (Fig. 1E), the broadening and pipette geometry artifacts are significantly reduced, and the images mirror the actual geometry to a much greater extent. The resolution improvement is quantified in Fig. 1F as FHWM/d_s_ (full width at half maximum (FWHM) divided by the diameter of the geometry), with an enhancement factor ((FHWM_SICM_/d_s_)/ (FHWM_SR-SICM_/d_s_)) for SR-SICM over SICM of 1.8–4.8 depending on the size of the imaged structure and the imaging set-point.

SICM image deconvolution is limited by user access to FEM software as well as the necessary computational power required to simulate reliable PSFs of pipettes. To avoid this disadvantage and make SR-SICM widely available, we have developed a software application for download and easy implementation. The application contains a library of FEM results obtained for pipettes with different geometrical shapes at different imaging conditions. Based on this extensive library, the PSF of custom pipettes can be estimated, followed by the input of SICM images and the acquirement of enhanced images using deconvolution algorithms. A detailed description of the program is found in Supplementary Fig. 2.

### Super-resolving basic and complex shapes

**Fig. 2:**
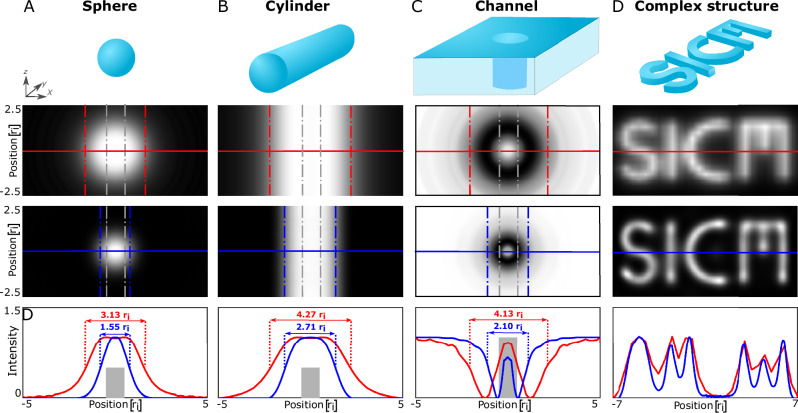
Super-resolving basic and complex geometrical shapes. The top row shows the various types of structures examined: **A** Sphere, **B** Cylinder, **C** channels and **D** “SICM” logo. The second row shows the simulated images produced by a standard SICM imaging approach, while the third row shows the super-resolved images produced by SR-SICM. The fourth row shows line profile comparisons for SICM (red) and SR-SICM (blue). The actual width of the geometries is highlighted by the gray stippled lines and boxes.

To further determine the capabilities of SR-SICM, a variety of basic shapes are imaged. Shapes such as spheres, cylinders and channels are studied as these approximate various important nanostructures such as nanoparticles^41^, protein fibrils^42^, biological pores and transport channels^43^. Structures that are smaller than the pipette opening with radius = *r*_i_/2 (Fig. 2) and radius = *r*_i_/5 (Supplementary Fig. 3) are studied as this allows greater understanding of the fundamental image improvements by SR-SICM. Protruding structures such as the sphere and a down-lying cylinder (Fig. 2A, B) appear wider than the actual geometry due to diffusional broadening, which is the most common SICM-related artifact^32^. Performing SR-SICM results in images that mirror the actual topography more faithfully. To perform a quantitative analysis, the FWHM is examined before and after deconvolution and compared to the width of the objects (gray stippled lines). For spheres, the FWHM reduces from 3.13 *r*_i_ to 1.55 *r*_i_, while for a down-lying cylinder it reduces from 4.27 *r*_i_ to 2.71 *r*_i_. For a channel, a ring-shaped profile is observed, which is commonly seen for sufficiently small structures such as point structure^32^ as also shown in Fig. 1. Here, a reduction in the FWHM of 4.13 *r*_i_ to 2.10 *r*_i_ is observed, while the ring-shape artifact is nearly removed. Similar gains in image resolution are also observed for even smaller structures with radius = *r*_i_/5 (Supplementary Fig. 3).

The imaging of holes and channels is of special interest, as most SPM techniques produce image artifacts due to the physical dimensions of the probe hindering access to the sample surface. Simulated SICM imaging of channels with different diameters shows strong image artifacts in the form of ring-shaped holes with a shallow depth (Supplementary Fig. 4). SR-SICM can remove the ring-shaped artifact for a channel with a radius of *r*_i_, but only limit it to a channel with a radius of *r*_i_/2. For simple sample structures SR-SICM will generally provide a quantitative improvement of resolution, while for sample structures larger than *r*_i_/2 it will also remove qualitative artifacts, such as the ring-shaped structures in Fig. 2C, Supplementary Figs. 3 and 4.

However, numerous non-organic and biological structures are significantly more structural complex and thus cannot be approximated by the basic shapes in Fig. 2A–C. To highlight the versatility of SR-SICM, structures with multiple complex geometrical features in a single image are studied in the form of an “SICM” logo (Fig. 2D). Here, it is seen that SR-SICM can achieve a high-resolution output of a complex image. The high-resolution capabilities of SICM are furthermore studied in detail in Supplementary Figs. 5 and 6, where the improvement in lateral resolution is quantified both theoretically and experimentally with good agreement. In both cases, the resolution is found to be improved from at least 3 *r*_i_–2 *r*_i_ (enhancement of 33%), depending on the imaging conditions.

### Super-resolving biological nanostructures

Nanoscale structural characterization of biomaterials and biomolecules is fundamental in order to understand their functional properties. Scanning probe methods, including AFM in particular are frequently utilized in the study of biomaterials as it uniquely provides real-time high-resolution imaging under native conditions^1^. However, AFM imaging requires physical contact between the tip and sample, which can easily deform or even damage biological materials and biomolecules, thereby interfering with the structure and dynamics. SICM circumvents this issue with its non-contact operation, which can be combined with its simultaneous surface charge mapping in addition to other surface sensing and manipulation capabilities. However, SICM suffers from a comparable worse lateral resolution in addition to numerous artifacts attributed to its non-contact operation^32^. SR-SICM is a novel super-resolution scanning probe technique that combines the advantages of SPM and super-resolution optical microscopy for achieving high-resolution imaging under non-contact conditions. To determine the real-world performance and versatility, this method is applied to experimental datasets containing biological nanostructures based on peptide, lipid, and DNA.

**Fig. 3:**
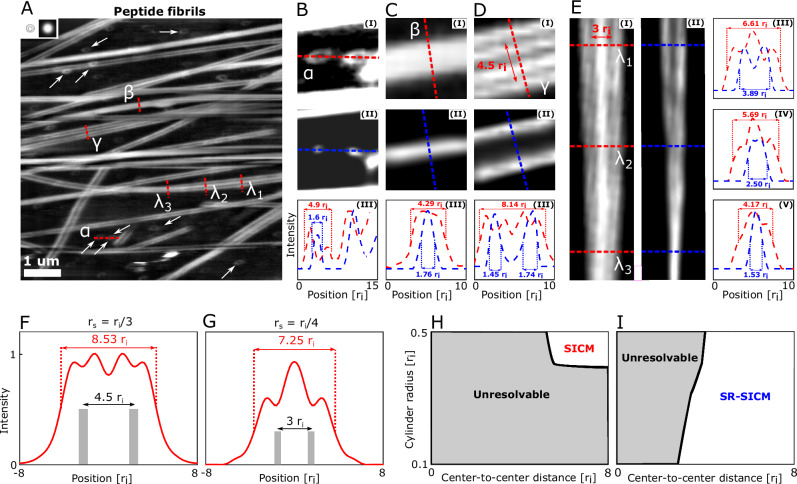
SR-SICM imaging of amyloid peptide fibers. **A** Experimental SICM image of a sample containing peptide fibrils. A pipette with an inner radius of 45 nm was used and the top-left inserts show the pipette tip schematic and calculated point-spread function, respectively. Four areas of interest are indicated, (*α*), (*β*), (*γ*) and (*λ*). These areas include a fibril particle (**B**), fibril (**C**), two parallel fibrils (**D**) and a branching fibril (**E**). For (**B**–**D**), subfigures (**I**), (**II**) and (**III**) are SICM, SR-SICM and line-profiles, respectively. For **E**, subfigures (**I**), (**II**) are the SICM and SR-SICM images, while (**III**), (**IV**) and (**V**) are line profiles of *λ*_1,_ *λ*_2_, and *λ*_3_. The line-profiles of *γ* and *λ*_1_ in (**D**, **E**) are reproduced theoretically by simulating line-profiles of two adjacent cylinders separated by a distance of 4.5 *r*_i_ (**F**) and 3 *r*_i_ (**G**). Graphical maps are produced exploring the imaging capabilities of (**H**) regular and SR-SICM (**I**) when imaging two adjacent cylinders with different radius and center-to-center distances. The colored areas correspond to geometries where imaged cylinders can be resolved according to the Rayleigh criterion while the striped areas correspond to geometries, which cannot be resolved.

Peptides and proteins are the most structurally complex and diverse set of biomolecules and consist of linked amino acids folded into function-dependent structures. Proteins that are misfolded are linked to a number of incurable degenerative diseases, such as Parkinson´s and Alzheimer’s^44^. KLVFF is a short amino acid sequence of the Alzheimer's responsible pathogenic amyloid-β (Aβ)^45^, and has been shown to be an inhibition component in the formation of toxic Aβ aggregates^46^. Furthermore, it has been shown to undergo nanoscale amyloid fibril formation under specific conditions^47^, which makes it a suitable test-sample for SR-SICM. Figure 3 shows the presence of multiple KVLFF peptide fibrils deposited on a mica surface.

Numerous areas of interest are chosen (denoted as *α*, *β*, *γ*, and *λ*) for further study to highlight the imaging capabilities of SR-SICM. Ring-like structures (*α*) are located numerous places in the SICM image in Fig. 3A (white arrows). Two of these structures (Fig. 3B**I**) are super-resolved simultaneously (Fig. 3B**II**). SR-SICM reveals that these are actually small fibril particles with sizes on the order of the pipette tip. These particles appear as rings due to pipette-sample convolution effects, where the structures mirror the pipette tip geometry. This effect is also theoretically predicted in Fig. 1 and is experimentally a commonly encountered SICM-related artifact^29^ when imaging structures compared to the size of the tip. Here, SR-SICM is capable of completely removing this artifact. Figure 3C**I** shows a single fiber (*β*) from the SICM image Fig. 3A with a FWHM of 4.29 *r*_i_. After applying SR-SICM (Fig. 3C**II**), over-broadening effects are revealed and the FWHM of the fibril is reduced to 1.76 *r*_i_. Figure 3D**I** shows what initially appears to be 4 individual fibrils in close proximity to each other (*γ*), while Fig. 3E**I** shows a fibril conformation that appears to consist of two small outer and one larger inner fibril that merge into one large single fibril. However, in both cases, SR-SICM (Fig. 3D**II** and Fig. 3E**II**) reveals that these fibrils have undergone a splitting artifact. Specifically, *γ* is shown to consist of two individual fibrils, while *λ* is two individual fibrils that merge into one. Splitting artifacts associated with the imaging of cylindrical objects comparable to the size of the pipette radius is also theoretically predicted and is noticeable for cylinders as large as *r*_cylinder_ = *r*_i_/2 (Fig. 2) and becomes more apparent for even smaller structures (Supplementary Fig. 3).

To further validate these conclusions, the line-profiles of *γ* and *λ*_1_ are reproduced with simulations. Here, two cylinders of various thickness and with a center-to-center distance of either 4.5 *r*_i_ or 3 *r*_i_ are studied (Supplementary Fig. 7). The resulting line profiles reveal good agreement between experimental and theoretical data (Fig. 3F, G), additionally verifying that SR-SICM correctly reveals two distinguishable cylinders.

To highlight the improvement of SR-SICM over SICM in the imaging of cylindrical objects, 2D graphical maps are constructed (Fig. 3H, I). These show the areas where two cylinders of various radii and center-to-center distances can be resolved according to the Rayleigh criterion. For regular SICM (Fig. 3H), this only corresponds to an area of approximately 10% (red area), which is due to the inherently low resolution and the emergence of splitting artifacts, which results in a single cylinder appearing as two distinguishable objects according to the Rayleigh criterion. However, SR-SICM (Fig. 3H), effectively removes this artifact as well as fundamentally improving the resolution and thereby allowing for 60% of the combinations explored to be resolved.

**Fig. 4:**
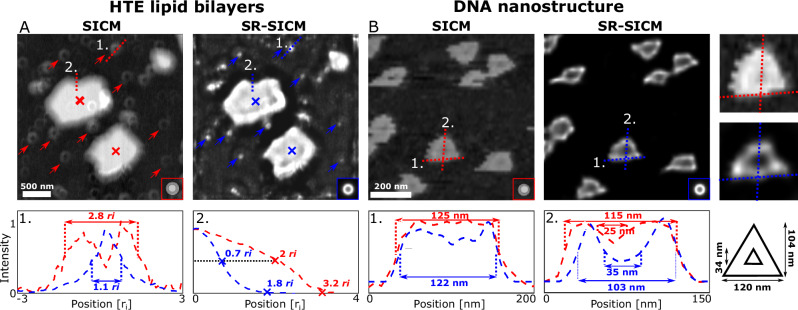
SR-SICM imaging on published datasets containing lipid bilayer and DNA origami nanoscale assemblies. **A** Heart total extract (HTE) Lipid bilayers deposited on a mica surface and scanned with a pipette with an inner radius of 74 nm. SICM image was provided by Dr. Johannes Rheinlaender and Prof. Dr. Tilman E. Schäffer from previously published data^29^. **B** Triangular DNA origami nanostructures containing a cavity scanned with a pipette with an inner radius of 16 nm. The SICM image was recorded by the authors and was previously published^55^.

A core strength of SR-SICM is its ability to super-resolve data post-acquisition. To highlight this capability, previously published SICM datasets containing supported lipid bilayers (SLBs) and DNA nanostructures are super-resolved. SLBs are commonly utilized as model membranes to gain insight into the fundamental physical and chemical properties of complex and dynamic cellular membranes^48,49^. Numerous SLB model systems have been studied in physiological conditions using SICM, ranging from lipid domains containing a single type lipid to biologically relevant multi-lipid model membranes^22,29,30^. An example is of Heart Total Extract SLB domains imaged on a mica surface (Fig. 4A)^29^. Here, two types of structures are present; SLB domains with sizes of several 100 nm (red crosses in SICM and blue crosses in SR-SICM image) and ring structures of approximately 70 nm in radius (red arrows in SICM and blue arrows in SR-SICM image). Applying SR-SICM to this image greatly mitigates the broadening artifacts of the lipid domain edge, which in reality is expected to be perpendicular to the support surface because of the close stacking of individual lipid molecules^50^. Additionally, as seen in the line profiles, SR-SICM reveals that observed ring structures are SICM artifacts that are in reality SLB domains with sizes on the order of the pipette (*r*_i_ = 74 nm). This demonstrates the future possibilities of super-resolving the complex composition of lipids in cellular membranes.

The unique programmability of DNA has allowed the assembly of static and dynamic nanoscale structures with sub-nanometer precision^51–54^. The Rothemund triangle^53^ (Fig. 4B^55^) has been harnessed as a molecular breadboard and drug nanocarrier^56,57^. The structure has outer sides that measure 120 nm, and a triangular cavity with sides of 40 nm. To quantitatively assess the improvement by SR-SICM, two lines are drawn on the SICM and SR-SICM images (Fig. 4B, top) with the corresponding line profiles in Fig. 4B, bottom. Line-profiles 1. examine the dimensions of the DNA origami outer sides, which measure 125 nm in the standard image, 122 nm in the SR-SICM image, while the real dimension is 120 nm. Line profiles 2. examine the height of the DNA origami and the size of the cavity. The height of the structure measures 115 nm in the SICM image, 103 nm in the SR-SICM image, while the actual height is 104 nm. The cavity, which in reality is 34 nm across, is just sufficiently resolved according to the Rayleigh criterion and measures only 25 nm in the SICM image. Applying SR-SICM, the cavity becomes resolvable well below the Rayleigh criterion and measures 35 nm. Importantly, the capability of SR-SICM to clearly resolve cavities or openings otherwise not easily resolvable demonstrates the future prospects of utilizing SR-SICM for mapping pores in the cell membrane.

## Discussion

Scanning ion-conductance microscopy is based on a true non-contact, non-invasive imaging methodology. While this is normally considered one of the key advantages of SICM, it becomes a limitation when pursuing super-resolution. The localization-based optical super-resolution methods rely on the photo-activation of a fluorophore and subsequent transition to an inactive state^7,8^, while localization AFM relies on spatial fluctuations of topographic features to improve the resolution^9^. While this is not a possibility for SICM, the unique shape of the pipette tip enables intriguing perspectives for image deconvolution. Dual-barrel pipettes are often used in hybrid SICM techniques^58^, but they have also been used for high-resolution imaging of biomolecules^59^. These more complex pipette tips potentially introduce more imaging artifacts, and we foresee that the presented image deconvolution approach based on simulated pipette tip sensing zones can be applied to a variety of pipette shapes for both the mitigation of artifacts and the improvement of resolution, increasing the number of applications of SR-SICM considerably.

The sensing zone introduced in this work represents an idealized case, where the pipette tip is radically symmetrical, and surface charges are neglected. A radial symmetry is normally assumed, based on the standard pipette tip fabrication approach^60^, but asymmetric tip shapes could also be implemented in the image deconvolution algorithms. Glass pipettes are known to carry a negative surface charge, which will affect the ionic flux profile^22^. It would also be possible to include surface charge in the image deconvolution, but the current algorithms are based on a dimensionless geometry, which would no longer be the case when surface charges are included. Furthermore, the effect of surface charge can normally be neglected for small bias potentials, and it was therefore not included in the present study.

## Methods

### FEM calculations

Finite element modeling was performed in COMSOL Multiphysics 5.4 (COMSOL AB, Stockholm, Sweden). A 3D finite-element geometry was designed and can be seen in detail in Supplementary Fig. 1. Simulations were performed by simultaneously solving the Poisson and Nernst–Planck equation. The ionic current was calculated by integrating the total current passing through the upper boundary inside the nanopipette. Boundary conditions can be seen in detail in Supplementary Table 1. Simulations were performed using a pipette with an inner half angle of 3° and an outer to inner tip radius (*r*_o_/*r*_i_) of 1.25 at an imaging set-point of 99%. The pipette PSF was simulated by scanning a cylindrical structure with radius of *r*_i_*/5* and constructing a 2D image by applying rotational symmetry to reduce computational resources. A custom MATLAB (MathWorks) script was written to simulate the image formation in an SICM setup. The script was run while enabling COMSOL Multiphysics 5.4 with MATLAB.

### SICM imaging

SICM imaging was carried out using a Bio-XE SPM system (Park Systems, Korea) mounted on a Nikon Eclipse Ti microscope (Nikon, Japan). The system has a decoupled x-y piezoelectric stage (100 × 100 µm) and uses a DLPCA-200 current amplifier (FEMTO, Germany). Ag/AgCl electrodes were fabricated by immersion of silver wire (0.025 mm, Goodfellow, UK) in 0.1 M NaClO for at least 30 min. Nanopipettes were fabricated from quartz capillaries with inner and outer diameter of 0.50 mm and 1.00 mm, respectively, using a P-2000 F-type puller (Sutter Instruments, US) with the following protocol: HEAT: 750/600, FIL: 4/3, VEL: 30/40, DEL: 150/135 PULL: 80/100.

### Preparation of amyloid-like fibrils

Amyloid-like fibrils were grown from the modified peptide, KLVFF-NH2 (CASLO ApS, Denmark), according to previously reported methods. The peptide was carefully suspended in NaOH (1 mg/mL, pH 12) and incubated for 24 h under quiescent conditions. Fibril samples were prepared by depositing 10 μM peptide solution on freshly cleaved mica and incubated 5 min before being blow dried with nitrogen.

## Supplementary information

Supplementary Information

## Data Availability

Data presented in this study is available at: https://zenodo.org/communities/bio-spm.

## Code availability

The software application for performing Super-Resolution SICM can be found at: https://github.com/Bio-SPM-Group/Super-Resolution-SICM-Application.
