# Supplementary material for: Super-resolution imaging with nanopipettes
Source: Npj Imaging. 2025 Sep 22;3:46. doi: 10.1038/s44303-025-00108-9 (PMC12454638; doi:10.1038/s44303-025-00108-9)
Supplement: Supplementary file 1 — Supplementary Information [file 44303_2025_108_MOESM1_ESM.docx]

**Supplementary information for:**

**Super-resolution imaging with nanopipettes**

Steffan Møller Sønderskov^1,2^, Lasse Hyldgaard Klausen^1^, Sebastian Amland Skaanvik^1,3^, Xiaojun Han^4,^* & Mingdong Dong^1,5,^*

1 Interdisciplinary Nanoscience Center (iNANO), Aarhus University, Aarhus, Denmark

2 Dansk Fundamental Metrologi - Danish National Metrology Institute, Hoersholm, Denmark

3 Department of Chemistry, Western University, London, Ontario, Canada

4 State Key Laboratory of Urban Water Resource and Environment, School of Chemistry and Chemical Engineering, Harbin Institute of Technology, Harbin, China

5 Laboratory of Biosensors and Bioelectronics, Institute for Biomedical Engineering, ETH Zurich, Zurich, Switzerland

E-mail: dong@inano.au.dk, hanxiaojun@hit.edu.cn

**Contents list:**

Figures S1-S7

Table S1

Supplementary note 1

**Supplementary information figure 1**


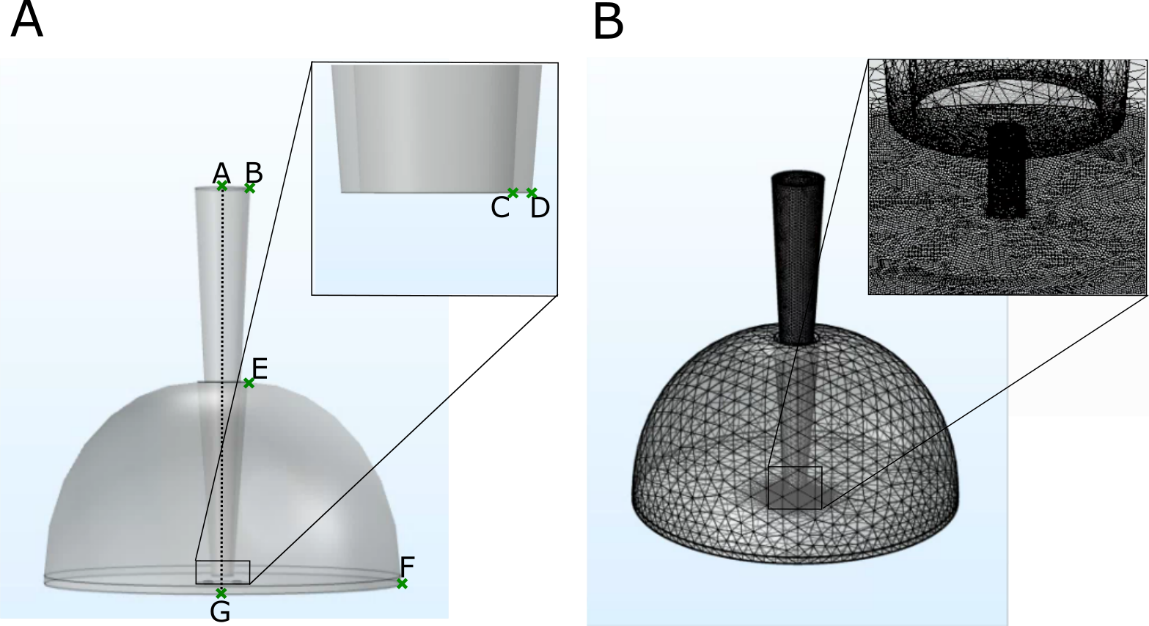


**Supplementary Figure 1. Finite-element geometry** View of the pipette geometry and mesh constructed for finite-element analysis of a single-barrel pipette. A small cylindrical structure acts as a point-emitter. Boundary conditions (letters A to H) are described in detail in supplementary table 1.

**Supplementary information figure 2**


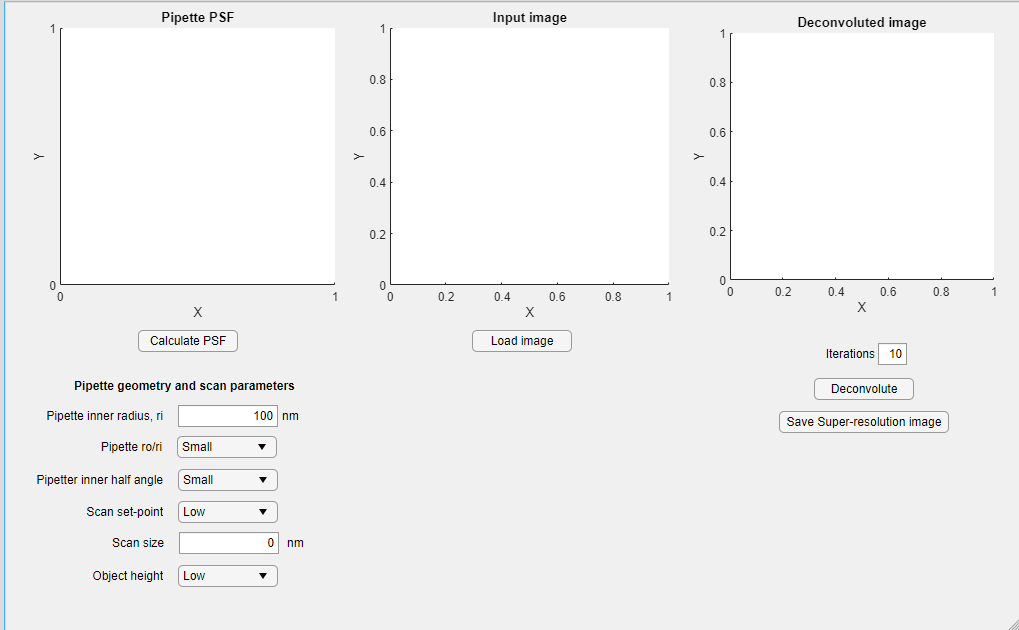


**Supplementary Figure 2. MATLAB application for super-resolution imaging** Panel 1 allows for the user to input basic pipette geometry and scan parameters for calculating an appropriate pipette point spread function (PSF). Panel 2 allows the user to load an SICM image. In Panel 3, the user can deconvolute the loaded image and thereby obtain a super-resolved image by first specifying # of deconvolution iterations (standard is 10). Afterwards, the super-resolved image can be saved.

**Supplementary information figure 3**


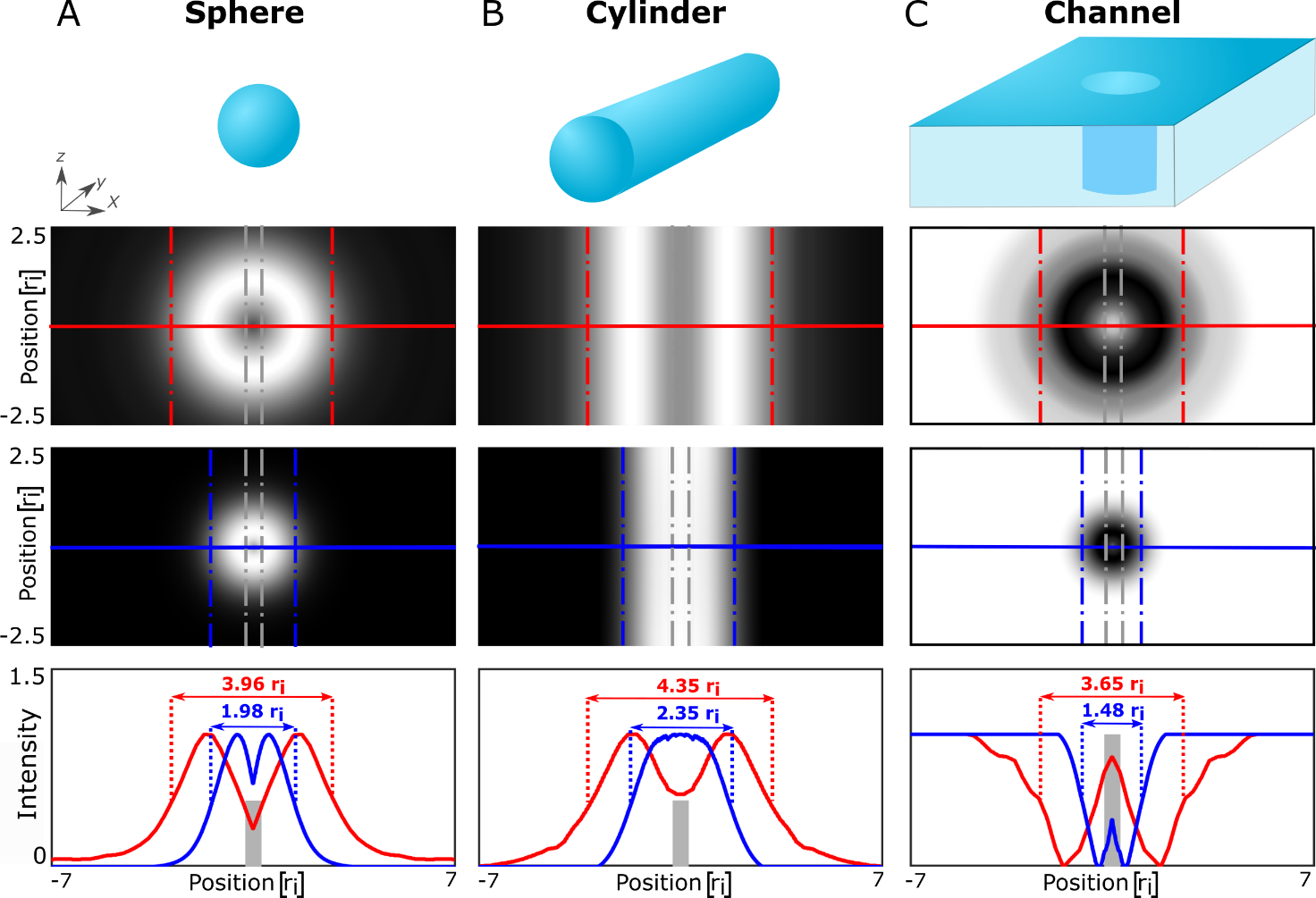


**Supplementary Figure 3 Super-resolving basic and complex geometrical shapes** The top row illustrates the various types of structures examined: (A) Sphere, (B) Cylinder and (C) channels. Second row is the imaging output and third row contains the super-resolved images accompanied by line profiles in fourth row for SICM (red) and SR-SICM (blue). The geometrical structures are highlighted in green. The actual width of the geometries is highlighted by the grey stippled lines and boxes.

**Supplementary information figure 4**


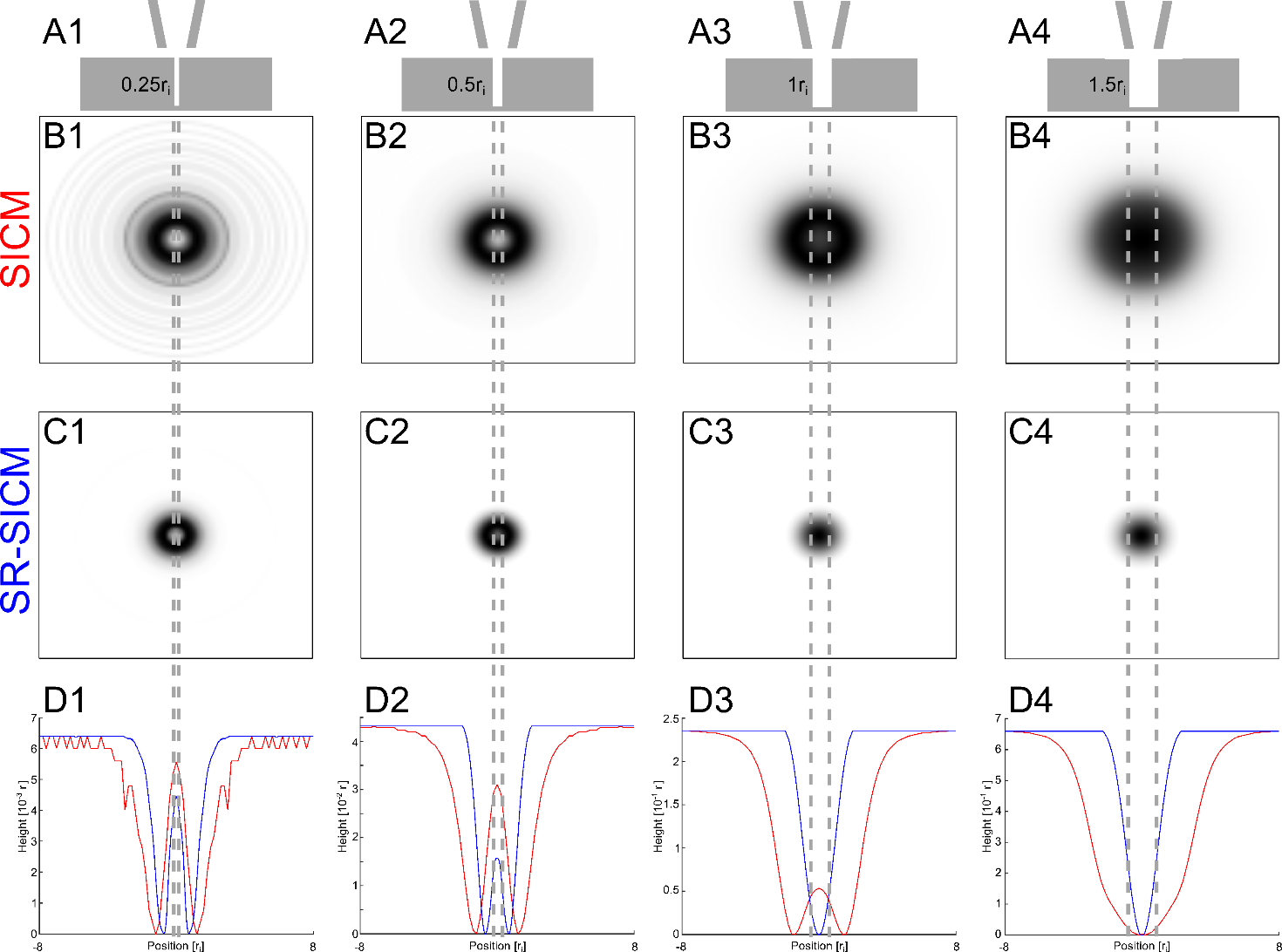


**Supplementary Figure 4. SR-SICM mitigation of artefacts when imaging holes.** Row (A) shows schematics of pipette tip and hole size used for FEM simulations. Hole radii of 0.25 (A1), 0.5 (A2), 1 (A3) and 1.5 (A4) times the pipette inner radius were used. Row (B) shows the simulated SICM images. Row (C) shows SR-SICM images obtained by image deconvolution. Row (D) shows line profiles through the middle of SICM (red lines) and SR-SICM (blue lines) images. The vertical grey dashed lines show the size of the hole structures.

**Supplementary information figure 5**

To estimate the fundamental lateral resolution, two cylindrical objects of diameter and height *h_0_* equal to the pipette inner radius, *r_i_* are considered. The value for fundamental lateral resolution is determined, when *d,* leads to fulfilment of the Rayleigh criterion i.e., features are resolved when a dip of 24 % of the profile maxima relative to the background is recorded. Four cases are of main interest; *d* = 1 *r_i_*, where the cylindrical structures are in contact, *d* = 2 *r_i_* which is the resolution achieved satisfying the Rayleigh criterion after performing deconvolution, *d* = 3 *r_i_*, the traditional resolution and, *d* = 4 *r_i_* where deconvolution yields fully distinguishable structures in contrast to 8 *r_i_* without deconvolution.

**
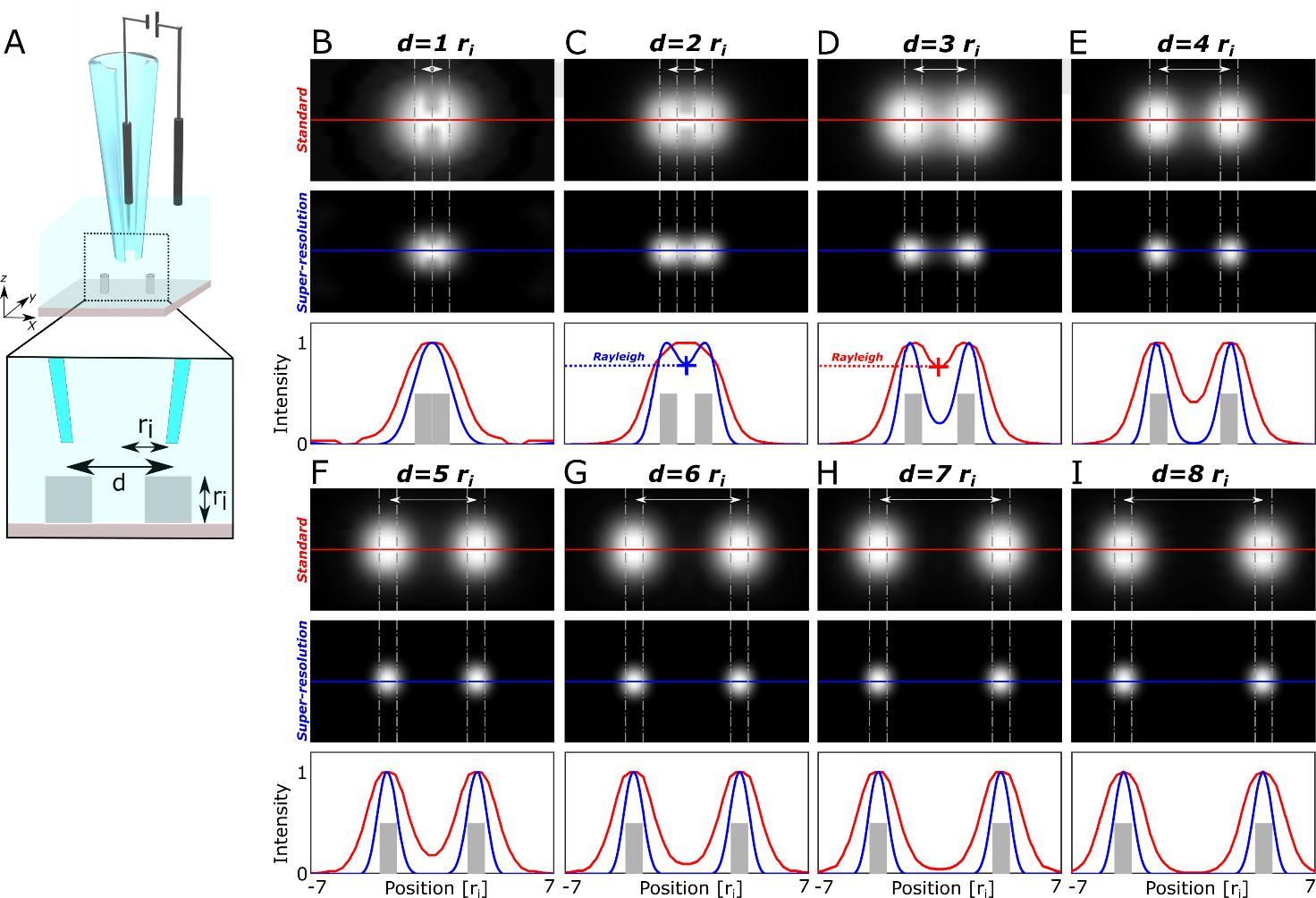
**

**Supplementary Figure 5. Determining the fundamental lateral resolution of SICM and SR-SICM** (A) Schematic showing the principle, where two cylindrical structures are placed with a center-to-center distance, *d.* (B-I) Full images accompanied by line-scans for *d* = 1–8 *r_i_.*

**Supplementary information figure 6**

**
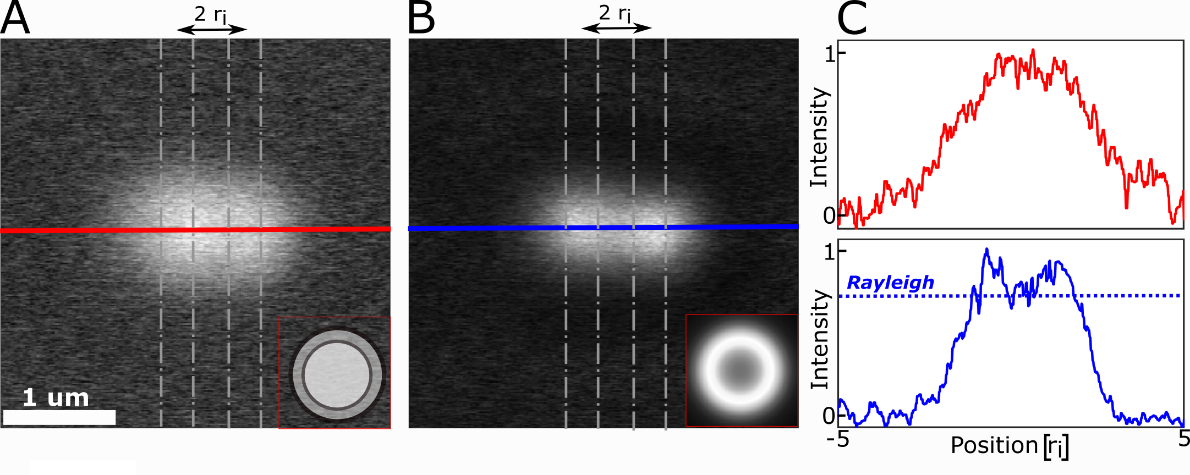
**

**Supplementary Figure 6. Experimental determination of the improvement in lateral resolution from SR-SICM.** (A) SICM image of two cylindrical particles (center to center distance of 2 *r_i_*) with height and width approximately equal to *r_i_*. These particles are not distinguishable according to the Rayleigh criterion. (B) SR-SICM of the same image showing that the particles are now easily distinguishable. The resolution of regular SICM is approximately 2 *r_i_*, thereby SR-SICM shows an improvement of at least 50 % in the fundamental lateral resolution. The image was obtained using a pipette with inner radius of 300 nm.

**Supplementary information figure 7**

**
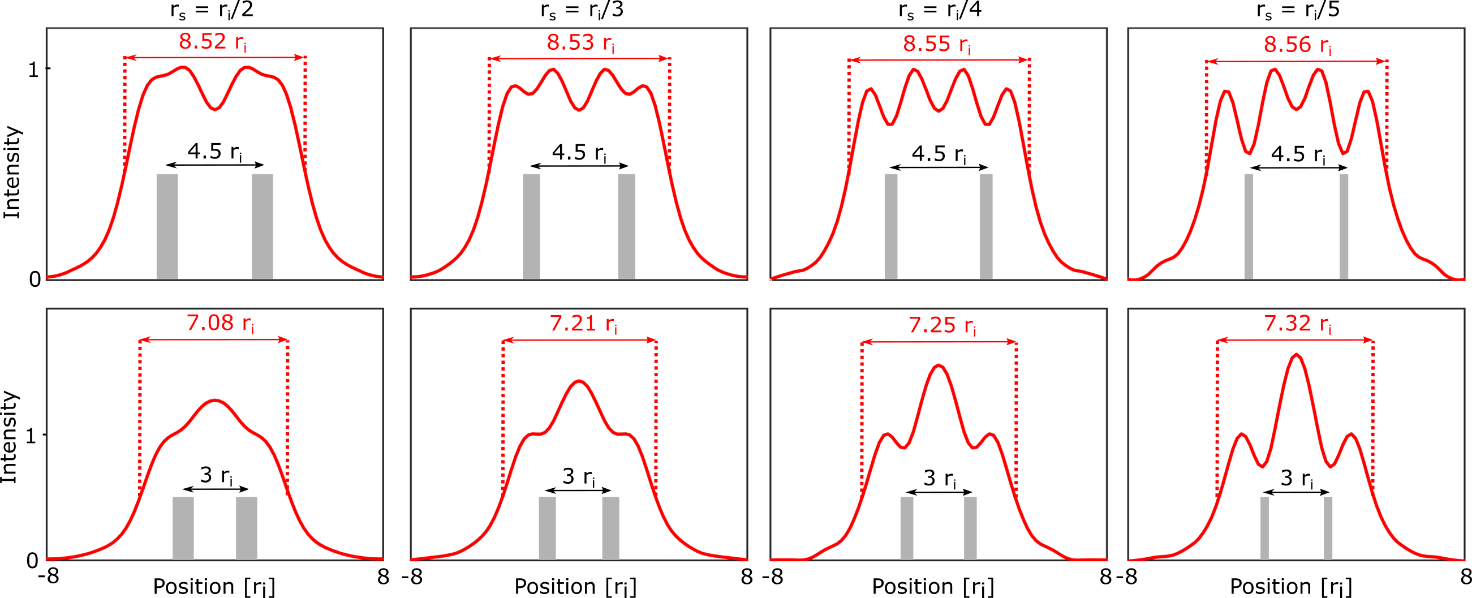
**

**Supplementary Figure 7. Imaging and SR-SICM of cylindrical structures with radius, r_s_.** SICM imaging and SR-SICM imaging of cylinders with radius (A) r_s_ = r_i_/4, (B) r_s_ = r_i_/2, (C) r_s_ = 3r_i_/4 and (D) r_s_ = r_i_.

**Supplementary table 1**

**Single-barrel pipette simulation boundary conditions:**

| Boundary | Description | Ion flow (NP) | Potential (P) |
| --- | --- | --- | --- |
| AB | Pipette top / electrode | Reservoir: *c_i_=c* | Electrode: *V=V_0_* |
| BC | Pipette inner wall | Isolating: *n*•*N_i_=*0 | *N/A* |
| CD | Pipette tip | Isolating: *n*•*N_i_=*0 | *N/A* |
| DE | Pipette outer wall | Isolating: *n*•*N_i_=*0 | *N/A* |
| EF | Water bath / electrode | Reservoir: *c_i_=c* | Electrode: *V=*0 |
| FG | Sample surface | Isolating: *n*•*N_i_=*0 | *N/A* |

**Supplementary table 1. Boundary conditions applied for simulations.** The boundaries (between letters A to G) are shown in Supplementary Figure 7.

**Supplementary note 1**

**Manual for SR-SICM**

**SR-SICM code**

1. Create geometrical point structure by running the code **point_structure.m**.
2. Run the file, **cylinder_scan.m**, which makes an SICM scan image of a cylindrical structure based on data obtained from FEM (**point_scan_data.m**).
3. Run the file, **deconvolution.m**, which first performs deconvolution based on the images obtained when running **point_structure.m** and **cylinder_scan.m** to produce an estimate of the PSF of the pipette. Next, deconvolution is performed on the PSF of the pipette and a test image (**data_structure.png**) consisting of two cylindrical structures in vicinity of each other. Finally, a super-resolved image is produced (**enhanced_image.png**).

**Testing SR-SICM app with published experimental data**

The capabilities of SR-SICM can be seen by performing super-resolution imaging on two SICM images that have been published in literature (DNA origami nanostructures and lipid-bilayer).

1. Install the MATLAB app, **SR-SICM_app.mlappinstall** from the MATLAB App folder.
2. Open the app and input the scan parameters from table 1 for either the DNA origami structure or Lipid bilayer and press the button, **Calculate PSF**.
3. Load either the DNA origami or Lipid bilayer image by pressing the button, **Load image** and locating the image in the **Experimental data** folder.

Tabel 1 Scan parameters

|  | DNA origami | Lipid bilayer |
| --- | --- | --- |
| Pipette inner radius, r_i_ (inner radius of the pipette orifice) | 1 nm | 1 nm |
| Pipette r_o_/r_i_ (ratio of the outer to inner radius of the pipette orifice), Small: 1.25, Large: 2 | Small | Small |
| Pipette inner half angle (Inner half angle of the pipette)  Small: 3°, Large: 6° | Small | Small |
| Scan set-point (SICM imaging scan set-point)  Low: 98.5 %, Medium: 99 %, High: 99.5 % | Medium | Low |
| Scan size (Size of the acquired SICM image) | 75 nm | 40 nm |
| Object height (Height of scanned object)  Low: r_i_/2, High: r_i_ | Low | High |
